# Sustainable development of China’s aesthetic teaching in long-term policy changes

**DOI:** 10.1371/journal.pone.0334315

**Published:** 2025-10-14

**Authors:** Xuebing Wang, Xiaohui Chen, Pengxi Qin, Jia Lu, Hanxi Wang

**Affiliations:** 1 School of Information Science and Technology, Northeast Normal University, Changchun, China; 2 School of Computer Engineering, Guilin University of Electronic Technology, Beihai, China; 3 School of Geographical Sciences, Harbin Normal University, Harbin, China; Universidade Federal do Tocantins, BRAZIL

## Abstract

The construction level of the teaching staff in aesthetic education determines the education quality, and relevant policies are the guarantee for the teaching staff construction. Due to multiple factors, the scientific formulation of aesthetic education policies in China is difficult and complex. The study objective is to enhance the role of policy in the teaching staff construction. This study was based on the theories proposed by MacDonald and Elmore, and 58 out of 1644 policy documents on the teaching staff construction in aesthetic education from 1978 to 2023 were selected to construct a two-dimensional analysis framework centered on policy instruments. The study indicated that the construction policy of China’s teaching staff in aesthetic education focused on professional development, the level of issue institutions was constantly improving, and policy coordination was constantly enhancing. The application ratio of different policy instruments was unbalanced. The content distribution of China’s aesthetic education policy was unbalanced. The selection of policy instruments by the Chinese government had a high degree of compatibility with policy content. This study provides reference for formulating scientific policies in the construction of the aesthetic education teacher team.

## 1. Introduction

Aesthetic education is the process of educating students to master aesthetic abilities and cultivate correct aesthetic education concepts [1]. Aesthetic education is an important means of cultivating people’s artistic cultivation and aesthetic ability, which has received widespread attention from the global education community in recent years. Culture and art are the most important components of holistic education, and comprehensive education ensures the full development of human personality including creativity, cognition, emotion, aesthetics, sociality, and spirituality [2]. Sustainable development goal formulated by the United Nations proposes the construction of quality education [3]. The quality education in China aims to cultivate the comprehensive development of people including moral, intellectual, physical, aesthetic, and labor [4]. Therefore, aesthetic education is an important content of quality education. The laws, formulated courses, and evaluation standards for all countries in the world have been promulgated to ensure the smooth development of aesthetic education. The “Goal 2000 - American Education Act” issued by United States included art as a core discipline in American primary and secondary schools, and National Standards for Art Education in the United States further made art education more standardized [5]. In addition, policies were also introduced to guide the development of aesthetic education in Germany, Japan, the United Kingdom, Canada, Australia, and other countries [6]. China’s education accelerates the development pace after the reform, which promulgates the “Compulsory Education Law” (1986), “Teacher Law” (1993), “Higher Education Law” (1998) and other educational laws [7]. With the acceleration of the education modernization process in China, the comprehensive promotion of quality education and the proposal of ideas such as “simultaneous development of five educations” have promoted the development of aesthetic education.

Aesthetic education was advocated by China not only to target art disciplines (music, dance, and fine arts) but also to cultivate students’ aesthetic abilities and creativity [8]. In recent years, multiple policies were successively introduced to guide the teaching staff construction of aesthetic education in China. In 2015, the General Office of the State Council of PRC issued the opinion on comprehensively strengthening and improving school aesthetic education work, which required the improvement of the overall quality of teaching staff in aesthetic education. The policy for the teaching staff construction in aesthetic education plays a crucial role in the sustainable development of aesthetic education [9]. Therefore, comprehensive analysis of the policy shortcomings is necessary.

Policy instruments are an effective subject of policy analysis and serve as a bridge between policy objectives and policy outcomes [10]. The use of policy instruments can analyze and organize the logic of the policy system [11]. At present, policy instruments are mainly applied in the fields of economics, education, medicine, etc., which provide a new perspective for scholars to find out the potential value of policy documents [12]. Earlier classifications of policy instruments tended to list policy types or categorize by subject area [13], and a few policy instruments (voluntary instruments, compulsory instruments, and mixed instruments) are widely used [14]. The theory of policy instruments was classified as compulsory instruments, incentive instruments, capability building instruments and learning instruments. The analytical dimensions of policy instruments involve with supply, environment, and demand. The command instruments, incentive instruments, capability building instruments and system changing instruments as policy analysis instruments have been proposed. The application of these policy instruments promotes the scientific formulation of policies [15].

With the gradual standardization and institutionalization of China’s teacher staff construction, relevant research on teacher staff construction is gradually comprehensive and systematic [16]. Policy instrument analysis has also been widely applied in the field of teacher staff building research [17]. China’s special education teacher policies have been analyzed using a full factor approach since China’s reform and opening up [18]. The 36 policy documents were quantitatively analyzed for the teaching staff construction to reveal important influencing factors in Chinese universities [19]. The historical context and evolutionary logic of the teaching staff construction in China’s rural areas was analyzed for policy optimization [20]. At present, research contents include early childhood teachers, university teachers, special education teachers, rural teachers, etc. [21].

The research on the construction of China’s aesthetic teacher staff mainly focuses on the development of rural aesthetic teacher staffs [22], the improvement of aesthetic teacher quality and ability, the expansion of professional training methods [23], and the reform of aesthetic education evaluation. So, there is a lack of systematic analysis of policy instruments for the aesthetic teacher staff. From the long-term development of the aesthetic teacher staff, this is not conducive to accurately grasping the path of aesthetic education construction. This study drew on the classification of policy instrument proposed by MacDonald and Elmore [24,25], which used command instruments, incentive instruments, capability building instruments, system changing instruments, and exhortation instruments as analytical dimensions. Command instruments enforce the implementation of aesthetic education curriculum standards or teacher qualification certification systems through regulations or administrative directives. Incentive instruments use economic incentives (such as special grants and performance rewards) to guide the development of aesthetic teachers. Capability building instruments enhance teachers’ abilities through aesthetic education teacher training programs. System changing instruments can delegate decision-making power in aesthetic education to local or school authorities. Exhortation instruments promote the values of aesthetic education by advocating or propagating guiding behavior. The application and existing problems of policy instruments in the construction of China’s aesthetic teacher staff was fully analyzed by artificial intelligence (AI) technology to promote the sustainable development [26,27]. Based on the previous discussions, this study raises the following research questions.

(1) What are the differences in policy analysis on the teaching staff construction in aesthetic education from different perspectives?
(2) What are the characteristics of policy instruments and content for the teaching staff construction in aesthetic education?
(3) What aspects of policy instruments are used to improve the construction quality of the teaching staff in aesthetic education?

## 2. Materials and methods

**Fig 1:**
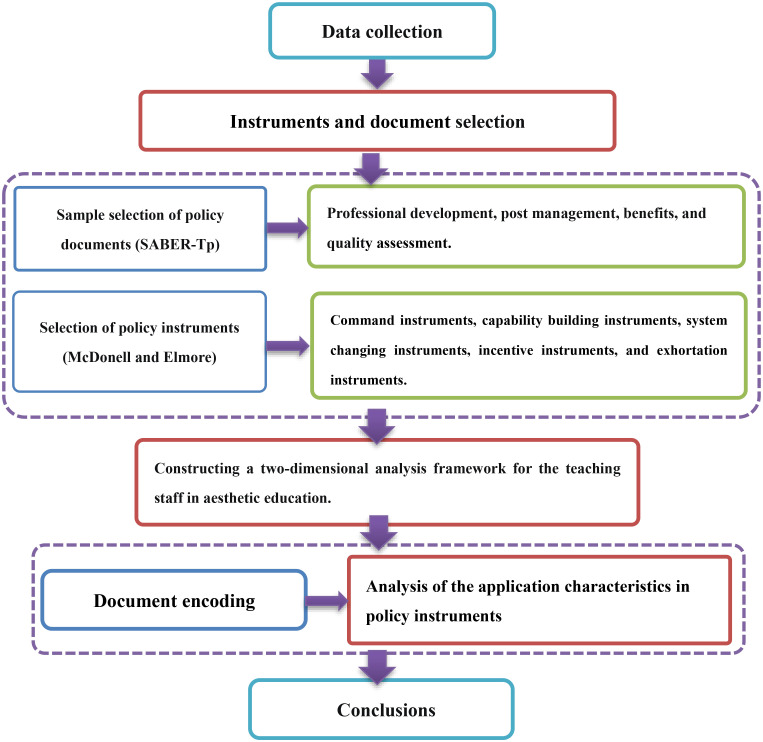
Research method flowchart.

Since the implementation of the reform and opening-up policy, the policy system for the construction of art education teachers in China has gradually taken shape. Relevant departments had successively issued a series of guided documents. Based on the analysis of policy texts, this study constructed a dual-dimensional analytical framework. The first dimension was the policy instrument dimension (X-axis), which focused on examining the types and characteristics of policy implementation methods. The second dimension was the policy content element dimension (Y-axis), which mainly analyzed the core constituent elements in the policy text. In the empirical research stage, the research team systematically extracted the relevant provisions for aesthetic education teachers from the selected policy samples and conducted structured coding processing using content analysis methods. The technical route and operational process of the research method have been shown in Fig 1.

### 2.1 Data selection

To systematically examine the evolution characteristics of the construction of aesthetic education teachers in China, this study had established a policy text analysis framework. At the level of keyword selection, “aesthetic education”, “aesthetic education teachers”, and “teacher construction” have been identified as the core search elements. The selection of policy texts followed a multi-dimensional screening principle. The policy authority standard focused on the level of the document issuer, with a particular emphasis on normative documents issued by the General Office of the Central Committee of the Communist Party of China (CCCPC), the State Council, and its affiliated ministries and commissions. The time dimension covered policy documents since the reform and opening up (1978 to the present), which fully presented the historical context of the construction of aesthetic education teachers. The text type included various policy carriers such as opinions, outlines, notifications, decisions, and regulations, which systematically reflected the combined application features of multi-dimensional policy instruments. The content dimension emphasized the integration of the professional characteristics of aesthetic education teachers and the common laws of teacher construction, which highlighted both the disciplinary specificity and the universal requirements for the construction of the teaching staff. Based on the above screening principles, the research team conducted a three-level screening of 529 policy texts from the State Council and 1,644 documents from the Ministry of Education and other ministries and commissions. The selection was mainly based on the keywords of “aesthetic education teachers” or “art teacher” in policy documents, which specifically involve the content of aesthetic education. The regulations for aesthetic education teachers were more specific. The 58 policy documents directly related to the construction of aesthetic education teachers as the core research samples were ultimately determined (S1 and S2 Tables). The research period started on April 10, 2022 and ended on May 28, 2024. The research method combining quantitative analysis and qualitative interpretation of policy texts was adopted to analyze the policy characteristics of the construction of aesthetic education teachers in China since the reform and opening up.

### 2.2 Analysis of the framework

This study was based on the policy instrument approach proposed by MacDonald and Elmore, as well as the analytical framework “Systematic Approach to Achieving Better Educational Outcomes - Teacher Policies” (SABER-Tp) developed by the World Bank in 2012 [28]. Subsequently, deeper into the combination of characteristics and change patterns of aesthetic teacher policy instruments were delved, and an analytical framework consisting of two dimensions: the policy instruments (X dimensions) and the policy content elements for aesthetic teachers (Y dimension) was constructed [29]. The application characteristics of different policy instruments in the X dimension were counted to analyze the functions and optimal application scenarios. Based on the four aspects (professional development, post management, benefits, quality evaluation) of the policy content for aesthetic teachers in the Y dimension, the frequency and distribution of the application of each policy content in policy instruments were calculated.

By using the classification method of policy instruments [30,31], a statistical analysis was conducted on the application frequency of educational policies related to aesthetic education. The five basic types of instruments (command-type, incentive-type, institutional reform-type, capacity-building-type, and exhortation-type instruments) formed the X-axis. The command-type instruments aimed to formulate corresponding rules to ensure behavioral norms. In China’s aesthetic education teacher policies, the command instruments manifested as the dual enforcement of laws and administrative instructions. For example, the “Law of the People’s Republic of China on Compulsory Education” clearly included aesthetic education in the legally prescribed curriculum, the “New Era Basic Education Strong Teacher Plan” of the Ministry of Education required that “regions lacking aesthetic education teachers should allocate a certain proportion each year for recruitment”, and strengthened the mandatory management of teacher staffing through methods such as “county-managed-school-employment”. Its mechanism effectively solved the “last mile” problem of the shortage of aesthetic education teachers in rural areas. At the same time, the capacity-building-type instruments aimed to provide funds, educational resources, professional training, and other supportive behaviors for educational activities. For example, the “National Training Program” and “Provincial Training Program” provided specialized training for rural aesthetic education teachers, which established of a collaborative training mechanism between universities and local governments. The institutional reform-type instruments aimed to achieve policy goals through organizational changes. In China’s aesthetic education teacher policies, the institutional reform instruments were manifested as the establishment of cross-departmental collaborative mechanisms, such as the joint release of the “Several Opinions on Promoting the Reform and Development of School Aesthetic Education” by the Ministry of Education, the Ministry of Culture, and the Ministry of Culture and Tourism. The incentive-type instruments aimed to promote the effective implementation of policies through positive encouragement. In China’s aesthetic education policies, the incentive instruments manifested as special subsidies for rural aesthetic education teachers, preferential treatment in professional title evaluation, etc., such as the “Three-Year Action Plan for School Aesthetic Education Teacher Allocation and Venue and Equipment Construction” of Huangshan City proposing “including the guidance of the second classroom undertaken by aesthetic education teachers in the workload and enjoying the same treatment in performance distribution”. Exhortation type instruments were those where the government conveys certain information for publicity, which internalized the sense of professional identity into the individual value pursuit of teachers with autonomy and value.

These five policy instruments were interrelated and had distinct essential differences. Particularly, the incentive instruments and the exhortation instruments stood out. From the perspective of behavioral-driven logic, the incentive instruments were assumed “economic man”, which believed that the behavioral choices of the teaching community were the rational calculation results of external stimuli and the maximization of individual interests. Their core mechanism was to directly change the cost-benefit structure of behavior through means such as bonuses, professional titles, and honors, which formed a linear causal chain of “behavior – reward”. For example, the incentive instruments transformed the professional identity of teachers into quantifiable external rewards by establishing the “Aesthetic Education Teaching Achievement Award” or giving preferential treatment to performance-based pay in the teacher policy, which drove teachers to actively increase teaching input. The exhortation instruments were based on the assumptions of “social man” or “cultural man”, which believed that the behavioral motivation stems from the internal recognition of teachers’ individual value beliefs or identity. Their action path did not rely on direct material compensation but rather build a meaning network through means such as policy discourse, value advocacy and exemplary demonstration, which stimulated the self-actualization needs and social responsibility of the target group. For example, the exhortation instruments attempted to internalize the professional identity of teachers into their value pursuit by emphasizing the educational mission of “educating through beauty” or the metaphor of “aesthetic education teachers were cultural ferrymen” in the aesthetic education teacher policy.

Based on the existing research, the content elements in the Y dimension were adjusted, and four content elements (professional development, job management, welfare benefits, and quality assessment) were determined as the Y-axis dimension of the analytical framework (S3 Table). Among them, professional development referred to the systematic process by which educators enhance their professional knowledge, teaching skills, and educational qualities through continuous learning, practice, and reflection. Job management was the management activity carried out by educational institutions through systematic classification, responsibility definition, and resource allocation, for planning, adjustment, and supervision of educational positions. Welfare benefits were the combination of economic and non-economic rewards provided by educational institutions or the government to educators. Quality assessment was a systematic activity that collects data on the educational process and results through standardized methods (such as examinations, observations, and questionnaires), which made value judgments based on the evidence.

Based on the two dimensions of basic policy instruments (X) and content elements (Y) of the teaching staff construction in aesthetic education, a two-dimensional analysis framework table of policies was constructed (Table 1). Policy instruments included command instruments, capability building instruments, incentive instruments, system changing instruments, and exhortation instruments. For each policy instrument, content keywords were constructed from four aspects (professional development, post management, benefits, and quality assessment).

**Table 1:** Two-dimensional analysis framework of policies on the teaching staff construction in aesthetic education.

Instrument (X)	Content (Y)
	Professional development, benefits, post management, and quality assessment.
Command instruments	Must, require, forbid, etc.
Capability building instruments	Training, improvement, education, etc.
Incentive instruments	Subsidy, reward, punishment, etc.
System changing instruments	Redistribution, reform, establishment, etc.
Exhortation instruments	Values, propaganda, guidance, etc.

### 2.3 Document code

The research method combining qualitative and quantitative analysis was adopted to systematically analyze the contents of policy documents. Based on the proposed analysis framework and samples, analysis units were defined, data categories were established, and policy documents were coded [31]. Based on the content analysis method, the policy contents related to aesthetic teachers were extracted, and the contents were coded according to the promulgation time of the policy documents. The information of “serial number - chapter - section - item” in the policy documents was extracted. Take the “National School Art Education Master Plan (1989 - 2000)” as an example, its release date was the earliest among the relevant policy documents, so its serial number was set as “1”. In this plan, the code “1 - 4 - 2–2” had a specific meaning. It precisely pointed to the second item under the second section of the fourth chapter of the plan, which focused on the construction of art education teachers. Based on this coding logic and method, the coding work was carried out for 58 policy documents related to the construction of art education teachers. Subsequently, a policy content analysis coding table with different hierarchical structures for the construction of art education teachers in China was constructed through systematic classification (S4 and S5 Tables).

## 3. Results

### 3.1 X-dimension index of policy instruments

**Fig 2:**
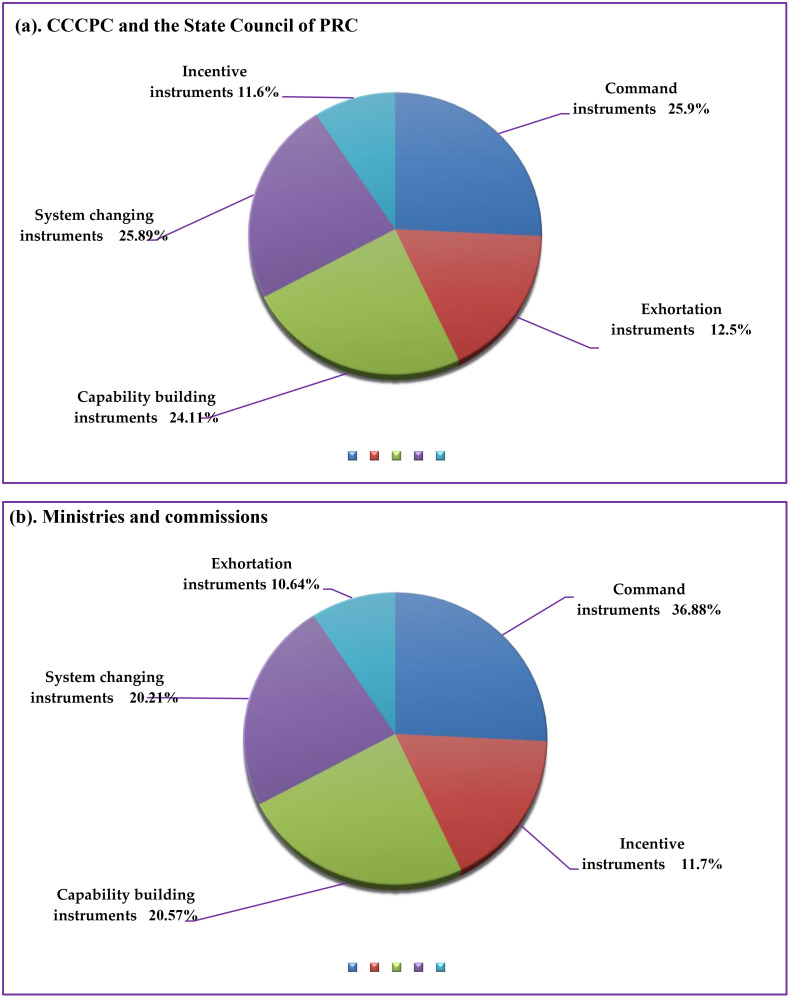
Policy instruments and content analysis on the teaching staff construction in aesthetic education (CCCPC, the State Council of PRC, Ministries and commissions including the Ministry of Education of PRC and Ministry of Culture and Tourism of PRC).

Various policy instruments were used to analyze the construction of the aesthetic teacher staff, which was divided into three levels (high, medium, and low) in terms of application frequency (Fig 2a). At the level of the CCCPC and the State Council of PRC, the highly frequently applied instruments were command instruments, which accounted for 25.9% (Fig 2a). The medium frequently used instruments were system changing instruments and capability building instruments, which accounted for 25.89% and 24.11% respectively (Fig 2a). The lowly used instruments were exhortation instruments and incentive instruments, which accounted for 12.5% and 11.6% respectively (Fig 2a). At the level of the Ministries and commissions including the Ministry of Education of PRC and Ministry of Culture and Tourism of PRC, the highly frequently applied instruments were command instruments (36.88%) (Fig 2b). The medium frequently used instruments were capability building instruments and system changing instruments, which accounted for 20.57% and 20.21% respectively (Fig 2b). The low frequently used instruments were incentive instruments and exhortation instruments, which accounted for 11.7% and 10.64%, respectively (Fig 2b).

**Fig 3:**
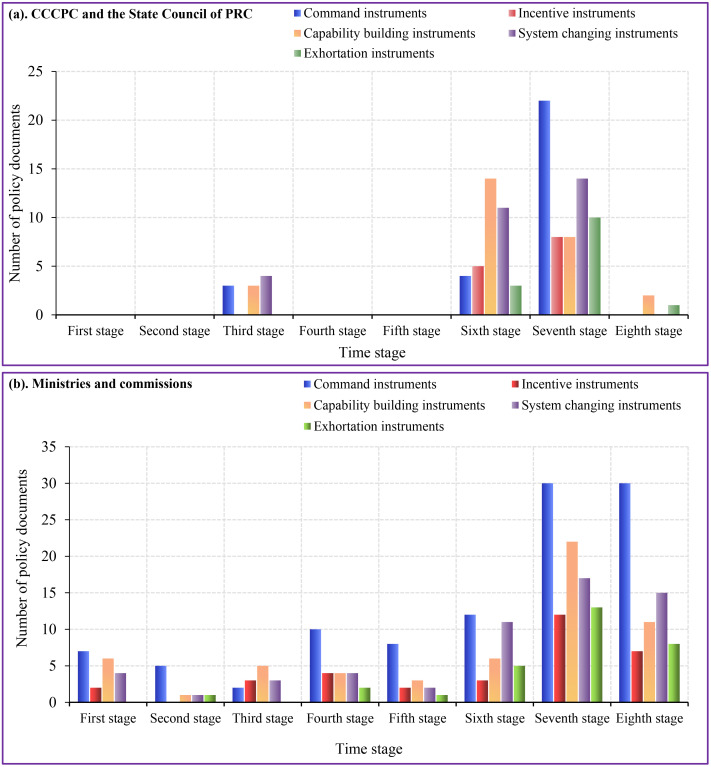
X.dimension time series of policy instruments for the construction of teaching staff in aesthetic education.

The statistical analysis results of the X-dimensional policy instruments for the construction of the aesthetic teacher staff are shown in Fig 3 and S6 Table. The differences between policy content command instruments, system changing instruments, and capacity building instruments at the level of the CCCPC and the State Council of PRC were relatively small. The policy content at the level of various ministries was mainly dominated by command instruments, which followed by capacity building and system changing instruments. There were fewer incentives and exhortation instruments. Based on the phased planning of China’s economic development (every 5 years) and highlighting the phased development characteristics of aesthetic education, this study was divided into eight stages. The first, second, third, fourth, fifth, sixth, seventh, and eight were 1986–1990, 1991–1995, 1996–2000, 2001–2005, 2006–2010, 2011–2015, 2016–2020, and 2021–2025.

Policy instruments at the level of the CCCPC and the State Council of PRC had been widely used in the sixth and seventh stages. The seventh stage had a higher number than the eighth stage among the exhortation instruments, but the proportion of exhortation instruments in the seventh stage (16.13%) was lower than that the eighth stage (33.33%) (S6 Table). The policy instruments of various ministries were relatively frequently applied in the seventh and eighth stages. Command instruments were most used in the last two stages. The second stage had the highest proportion of command instruments (62.5%) compared to the seventh stage (31.91%) and eighth stage (42.25%) (S6 Table).

### 3.2 Y-dimension index of policy contents

**Fig 4:**
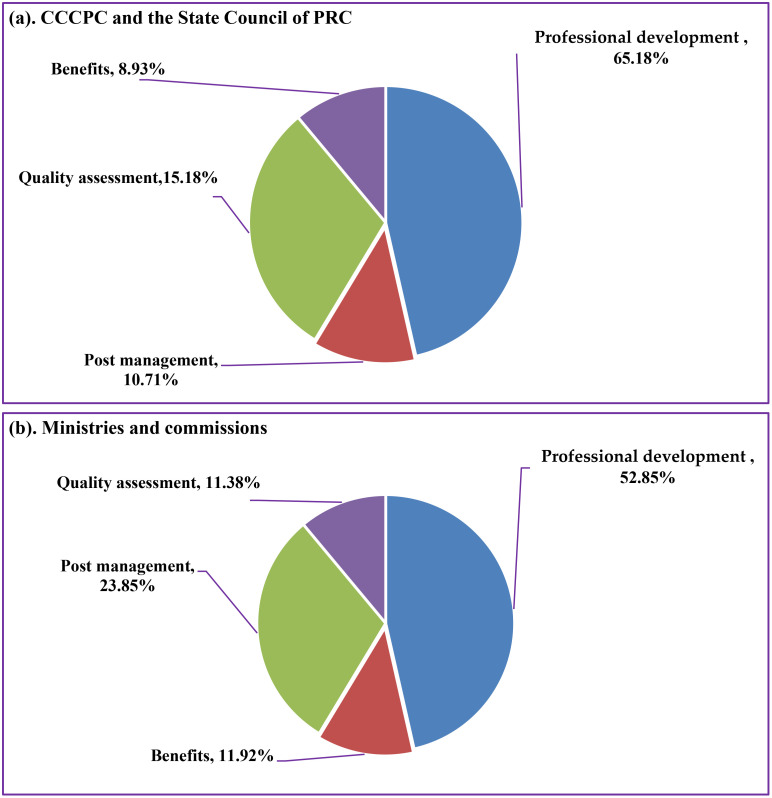
Policy content on the teaching staff construction in aesthetic education.

The policy contents included four dimensions (professional development, benefits, post management and quality assessment). Policy content on the teaching staff construction in aesthetic education is shown on Fig 4. At the level of the CCCPC and the State Council of PRC, the professional development of aesthetic teachers accounted for the largest proportion (65.18%) (Fig 4a), and the second was quality assessment (15.18%) (Fig 4a). Policies and regulations related to post management and benefits accounted for 10.71% and 8.93% respectively (Fig 4a). There were significant differences in China’s level of attention to various aspects of the teacher staff construction in aesthetic education. The policy documents from ministries and commissions had the highest proportion of content related to the professional development of aesthetic teachers (52.85%), which was followed by post management (23.85%) (Fig 4b). The relatively few policies and regulations related to quality assessment and benefits (11.92% and 11.38%) (Fig 4b). There were differences in the attention paid to policy content at different levels.

**Fig 5:**
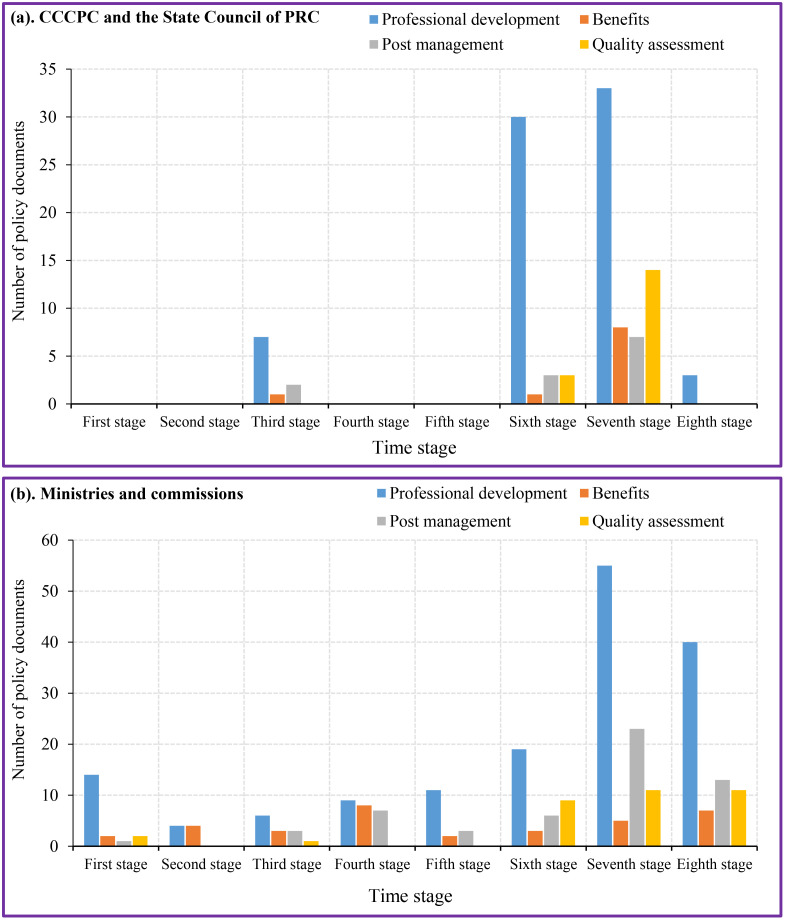
Y-dimension time series of policy contents for the construction of teaching staff in aesthetic education.

In terms of time series, the main line of professional development was always upheld in the China teacher staff of aesthetic education, which was the core of the aesthetic education staff construction at different stages (Fig 5a). Since the 12th Five Year Plan (2011–2015), China’s aesthetic teacher education had entered a period of rapid development year by year. The development of aesthetic teacher education in China during this period was manifested in the broadening of channels for cultivating teachers and the diversification of teacher development. Many comprehensive universities had established educational majors to expand the source of teachers and the singularity of teacher training programs. The CCCPC and the State Council of PRC provided guidance for the development of teachers based on their actual development situation. Multiple policy contents for aesthetic teachers had shown a continuous upward trend from the sixth stage to the seventh stage.

The Y-dimensional time series analysis results of the policy content for the construction of aesthetic teacher staff at the levels of the CCCPC, the State Council of PRC, the Ministry of Education of PRC, and other ministries are shown in Fig 5b and S7 Table. The policy content of the aesthetic teacher staff construction at the level of the Ministry of Education of PRC and other ministries was more diverse than that at the level of the CCCPC and the State Council of PRC. Professional development and benefits were reflected in each stage and adjusted according to the actual situation of each stage. The policy content of professional development was the most reflected in the 2016–2020. Post management was reflected in all other stages except for the second stage (1991–1995), and the policy content mentioned in the seventh stage (2016–2020) of post management was the most. At different stages, quality assessment was the least among the four policy contents, and it was in a missing state in the second, fourth, and fifth stages. However, the importance of quality assessment policy content has been reflected since the sixth stage. The policy on the teaching staff of aesthetic education is completed and unified during 2010–2015, 2016–2020 and 2021–2025, which shows an increasing trend year by year. Although teacher professional development presented the most content in the seventh stage, the proportion of aesthetic teacher professional development in the first stage was the highest (73.68%) (S7 Table).

### 3.3 Two-dimensional index of policy instruments and contents

This study conducted a cross analysis between the five policy instrument types in the X dimension and the four indicators in the Y dimension to obtain a two-dimensional analysis table of the policy document for the construction of the aesthetic teacher staff at the central level of the Communist Party of China (Table 2). The application proportion of policy instruments in professional development was capability building instruments (21.43%), system changing instruments (16.07%), command instruments (11.61%), exhortation instruments (11.61%), and incentive instruments (4.46%) (S4 Table). The application proportion of policy instruments in benefits was incentive instruments (6.25%), command instruments (1.79%) and capability building instruments (0.89%) (S4 Table). The application proportion of policy instruments in post management was system changing instruments (3.57%), command instruments (3.57%), capability building instruments (1.79%), incentive instruments (0.89%), and exhortation instruments (0.89%) (S4 Table). The application proportion of policy instruments in quality assessment was command instruments (8.93%) and system changing instruments (6.25%) (S4 Table).

**Table 2:** Two-dimensional analysis of the policy documents on the construction of teaching staff in aesthetic education (%).

CCCPC and the State Council of PRC						
Policy content	Policy instruments					
	Command instruments	Incentive instruments	Capability building instruments	System changing instruments	Exhortation instruments	Total
Professional development	13	5	24	18	13	73
Benefits	2	7	1	0	0	10
Post management	4	1	2	4	1	12
Quality assessment	10	0	0	7	0	17
**Ministries and commissions**						
Policy content	Policy instruments					
	Command instruments	Incentive instruments	Capability building instruments	System changing instruments	Exhortation instruments	Total
Professional development	42	14	52	27	23	158
Benefits	18	13	1	0	2	34
Post management	23	6	5	17	5	56
Quality assessment	21	0	0	13	0	34

The two-dimensional analysis of the policy document on the construction of the aesthetic teacher staff at the level of the Ministry of Education and other ministries is shown in Table 2. Capacity building instruments (18.44%) and command instruments (14.89%) were mainly used to promote professional development, while system changing instruments (9.57%), incentive instruments (8.16%) and exhortation instruments (4.96%) were less used (S5 Table). The main instruments used to improve benefits were command instruments (6.38%), incentive instruments (4.61%), exhortation instruments (0.71%) and capacity building instruments (0.35%) (S5 Table). Post management mainly used command instruments (8.16%) and system changing instruments (6.03%), while incentive instruments (2.13%), capacity building instruments (1.77%) and advisory instruments (1.77%) had a relatively low frequency of application (S5 Table). The main use of quality assessment was command instruments (7.45%) and system changing instruments (4.61%) (S5 Table).

## 4. Discussion

### 4.1 The change impact of policy documents number at different stages on aesthetic education

**Fig 6:**
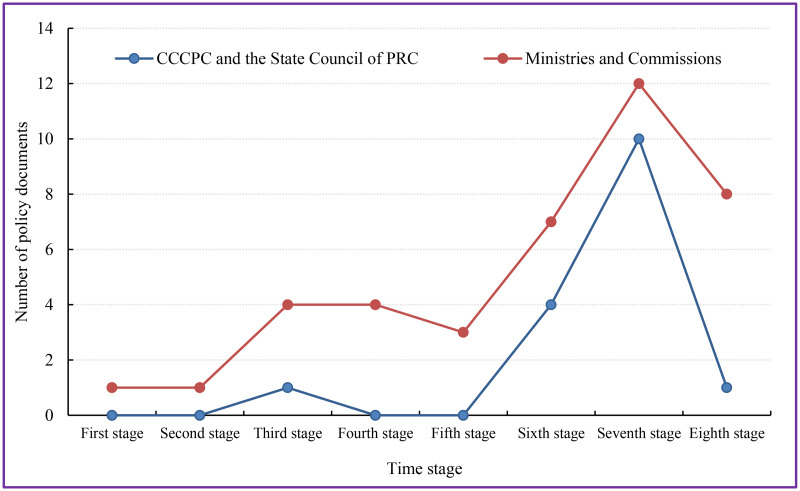
Number of policy documents related to aesthetic education.

The number of policy documents related to aesthetic education at different stages is shown on Fig 6. The number of publications by institutions at the State Council of PRC and above reached its peak in the sixth and seventh stages. China’s educational goals gradually shift from vague to specific and from single to comprehensive with the progress of society [32]. In 1986, the State Council of PRC announced the “Seventh Five Year Plan”, which stated that “all levels and types of schools should implement the policy of comprehensive development of moral education, intellectual education, physical education, and aesthetic education”. In April 1986, the “Compulsory Education Law of the People’s Republic of China” was issued, and compulsory education provided students with moral, intellectual, physical, aesthetic, and labor education. The “Compulsory Education Curriculum Plan and Curriculum Standards (2022 Edition)” issued by the Ministry of Education officially designated aesthetic education as a national level curriculum, which marked a new stage in the development of aesthetic education in China [33]. The number and importance of aesthetic education policy documents had increased significantly since the 18th National Congress of the Communist Party of China [34].

### 4.2 Hierarchy analysis of policy issuing institutions

The level of issuing institutions on aesthetic education policies had been continuously improved, and the policy coordination had been enhanced. The issuing institutions of aesthetic education policies were multi-level and diversified, which reflected the trend of collaboration and coordination among different departments. The “Opinions on Comprehensively Strengthening and Improving Aesthetic Education in Schools” issued by the State Council of PRC in 2015, which indicated that all localities established a new mechanism for colleges and universities to cooperate with local governments, industrial enterprises, and primary and secondary schools to cultivate aesthetic teachers. China had also begun to comprehensively call on different institutions to promote the rapid development of aesthetic education, which improved the systematic construction of the aesthetic education staff.

This had the same development trend as the United States in the development of aesthetic education related work. The research on the work of United States policy publishing agencies and art education mainly came from the US federal government and its affiliated departments, local art institutions and committees, for-profit organizations, non-profit organizations, foundations, research centers, and various schools [35], so the United States was broad and inclusive in the development of art education. The entire tax system and art policies in the United States were closely intertwined, which improved the speed and policy execution of aesthetic education development.

### 4.3 Analysis of policy instruments

Since the reform, the policy instruments adopted by the Chinese government had shifted from single to diverse, which was even more evident in the policies issued by various ministries and commissions. After 2000, five different types of policy instruments were widely used, which gradually matured in their application. Different types of policy instruments had different application scenarios, and the comprehensive use of multiple policy instruments enhanced their application effectiveness [36]. There was a certain imbalance in the number and proportion of policy instruments. Looking at the use of policy instruments in China’s aesthetic education policies since the reform, the CCCPC and the State Council of PRC had dominated by command instruments, system changing instruments, and capacity building instruments. The Ministry of Education of PRC and other departments mainly use command instruments, which are followed by capacity building and system changing instruments. This was related to China’s national conditions, which influenced by economic development and people’s living standards. Based on system control theory analysis, policy instruments were utilized to achieve standardized management of aesthetic education. Based on constructivist learning theory analysis, the use of capacity building instruments was beneficial for achieving personalized creative ability enhancement. As a complex adaptive system, the transformation of the aesthetic education system needed to follow Holland’s principle of “emergence” and achieve a dynamic balance of “cognition practice evaluation”. Although the differences in the application of policy instruments were reflected in the difference issues that needed to be addressed during specific historical periods, the imbalance in the proportion of policy applications was not conducive to the sustainable development of the teaching staff. The ideal application scenario for commanding instruments was clear policy objectives, clear means, relatively consistent goals among various levels and departments of the government, and sufficient resources for policy enforcement agencies [37]. Although there had been significant progress in the expansion of China’s aesthetic education resources [38], the conditions to apply more command instruments still need to be improved.

The serious shortage of incentive instruments in use made it difficult to stimulate the enthusiasm of teachers and schools to carry out aesthetic education, which limited the effectiveness of promoting aesthetic education. According to self-determination theory, the application of incentive instruments needed to meet the three elements of autonomy, competence, and belonging proposed by Desi, and achieved a “challenge success” experience loop through personalized difficulty adjustment. Insufficient use of exhortation instruments hindered teachers’ understanding and value experience of the connotation of aesthetic education in China, which affected the practical effectiveness of aesthetic education. According to the theory of emotional persuasion, the application of exhortation instruments needed to meet Petty’s ELM model. When the aesthetic object had a high emotional arousal, integrating value guidance into the process of art appreciation encouraged individuals to actively adjust their aesthetic cognitive structure.

### 4.4 Policy content analysis

Since the reform, the content of policies related to the professional development of aesthetic teachers had been at the forefront in different historical periods, and the frequency of application had also rapidly increased in the past decade. In addition to specific requirements for the development of the ability of the aesthetic teacher staff, relevant policy documents also proposed practical and feasible professional development plans based on the development background of the times [37]. How to improve the ability and quality of aesthetic teachers was also an important issue of concern for developed countries led by the United States.

The reform of aesthetic education evaluation was an important component of school aesthetic education work in the new era [39]. With the increasing number of aesthetic teachers, China had gradually begun to attach importance to establishing evaluation standards for aesthetic teachers and aesthetic education systems after entering the sixth stage. During this period, the policy content related to the quality evaluation of aesthetic teacher staff construction had significantly improved in terms of its application quantity at different levels. Quality assessment accounted for a relatively small proportion in policies, and aesthetic education evaluation had not received due attention in academic research related to aesthetic education [40]. The United States had more targeted and efficient implementation of art education standards. The Professional Standards for Art Teachers in the United States were a series of standards issued by the National Art Education Association, and art education and teacher standards formulated and promulgated by each state. They included the National Standards for Art Education, the Standards for Preparatory Art Teachers, the Professional Standards for Visual Art Educators, and corresponding state standards [41]. The establishment and flexible implementation of professional standards for art teachers effectively enhanced the professional quality of American art teachers. The basic standard was based on teacher competence standards and national standards in China. This approach further ensured education fairness, but poor flexibility made it difficult for unified standards to meet national requirements.

Benefits were an important issue that was not avoided in the teaching staff construction. Benefits were an important support for improving the overall professional quality of the aesthetic teacher staff and ensuring the development of the teacher staff, which had a relatively small proportion in policy provisions, and their proportion had shown a decreasing trend year by year over a period. Although its proportion had increased in the 14th Five Year Plan, it was generally at a relatively low level. Benefits were still a key issue restricting the aesthetic teacher staff construction, which were also the main reason for widening the education gap between urban and rural areas. South Korea also faced a serious shortage and loss of rural aesthetic teachers. To solve this problem, the South Korean Ministry of Education and Human Resources had expanded enrollment in 11 universities in the central and southern regions, which provided subsidies to teachers [42].

Post management was an important instrument to ensure the sustainable development of China’s aesthetic teacher staff, which had also increased in frequency over the past decade. Post management mainly achieved the overall goal of sustainable development by regulating teacher staffing, teacher allocation, and supplementary mechanisms. General subject teachers in South Korean served as class teachers and taught courses such as arithmetic, traditional culture, ethics, art, physical education, etc. General subject teachers solved the problem of insufficient aesthetic teachers in rural primary schools. The adoption of a “staffing of government affiliated institutions” teacher recruitment model in Chinese public schools had increased the attractiveness of outstanding talents. It greatly reduced the crisis among teachers and was not conducive to the sustainable development of the teaching staff in the long run. Canadian universities had formed a staff of reserve teachers, who chose those with better conditions to hire. Previously hired teachers faced a crisis of dismissal, but this model encouraged teachers to constantly supplement new professional knowledge, update teaching methods, and enhance their self-awareness and sense of crisis [43].

### 4.5 Two-dimensional analysis of policy instruments and contents

A two-dimensional analysis of policies related to the construction of the aesthetic teacher staff helped clarify the relationship between policy content and policy instrument usage strategies. In the research on policy instruments of the education field, researchers focused on rural teachers [44], labor education [45], physical education, and others. This study analyzed the policies related to the construction of China’s aesthetic teacher staff from the perspective of the corresponding relationship between policy content and the types of policy instruments. Overall, the selection of policy instruments was highly consistent with the policy content. In complex field problems, adopting a mixed approach of multiple policy instruments fully leveraged the effectiveness of different instruments. Benefits mainly used command and incentive instruments to ensure the implementation of policies. The key to enhancing the efficiency of policy implementation in the field of benefits was to use command instruments as the main body with incentive instruments. In terms of quality evaluation, command instruments were used to ensure execution and continuously improve institutional mechanisms with the help of system changing instruments. In terms of professional development, too much attention was paid to capacity building. The key role of motivational and advisory instruments had been overlooked, which made it difficult to stimulate the autonomy of aesthetic teachers in improving their professional abilities and levels. In terms of professional development and post management, the overuse of command instruments to a certain extent limited the internal driving force of the aesthetic teacher staff construction. Relying on advisory and incentive instruments was more likely to stimulate the endogenous power of aesthetic teachers.

## 5. Implications, limitations and future research focus

### 5.1 Implications

Chinese government had fully utilized various policy instruments to improve the quality of aesthetic education [46]. Aesthetic education covered multiple stages and fields, and the construction of the aesthetic teacher staff was also a complex issue [47]. There was a diversity of collaborative entities in the teaching staff construction in China. Extensive cooperation provided more positive and effective solutions to the problems. The relevant policy documents explained how to strengthen local coordination [48]. Guiding social funds, attracting social donations, and other multi-channel measures were aimed at promoting the interaction and interconnection between school resources and social resources. To promote the sustainable development of the aesthetic teacher staff, it was particularly important to strengthen multi-level and cross departmental cooperation among the government.

The teaching staff construction in aesthetic education also had a certain development law and cycle [49]. The construction of China’s teaching staff in aesthetic education had gone through stages such as quantity expansion and quality improvement There were significant differences in the types of policy instruments in different stages. The formulation of policies followed the construction laws of the teaching staff in aesthetic education and the growth of teachers.

The Education Informatization 2.0 Action Plan indicated that the “AI + Teacher Staff Construction Action” was launched to promote a new path of AI supporting teacher governance, teacher education, and education teaching [50]. Sharing of high-quality online resources had been adopted to address the shortcomings of aesthetic teachers [51]. When the number and quality of aesthetic teachers did not meet the needs of aesthetic education development, the use of information technology teaching methods radiated the courses of excellent aesthetic teachers to a wider region.

### 5.2 Limitations of research and future research focus

The focus of this study was on policies issued by the central government and various ministries, while policies issued by local governments were very few considerations. Local policies were the interpretation and supplement of relevant central policies based on local characteristics. Relevant research had conducted instrument analysis on local policies in other fields of education [52–54], but there was still a gap in research on the aesthetic teacher staff construction. This study will gradually incorporate local relevant policies in future work, a research route of “centralization before decentralization” will be adopted, the policy instrument of the central and various ministerial policies will be prioritized for analysis. China’s requirements and construction path for the construction of the aesthetic teacher staff will be explored at the macro level.

## 6. Conclusions

This study designed an “X-Y” two-dimensional analysis framework based on the perspective of policy instruments to analyze 58 policy documents related to the construction of the aesthetic education teacher team issued by China since the reform and opening up. The main research showed that the number of policy documents related to the construction of China’s aesthetic teacher staff was consistent with the changes in understanding of aesthetic education. The level of publishing institutions for aesthetic education policies was constantly improving, joint publications by multiple ministries were becoming more frequent, and the coordination of policy content was constantly improving. The multiple policy instruments were applied to achieve expected policy goals in Chinese government, but there was still a problem of proportion imbalance. There were significant differences in the proportion of different policy instruments. The imbalance in the proportion of policy applications was not conducive to the sustainable development of the teaching staff. Professional development has always been regarded as the main line of building a team of aesthetic education teachers in China, and other related content was mainly aimed at serving the professional development of teachers. Aesthetic education evaluation was still a weak link in the education evaluation system. The function of maintaining the sustainable development of the aesthetic teacher staff was limited. The selection of policy instruments by the Chinese government had a high degree of compatibility with policy content, but there was still a certain imbalance in the application frequency of specific policy instruments. In the future, this study will gradually incorporate local relevant policies, and the policy instrument of the central and various ministerial policies will be prioritized for analysis.

## Supporting information

Appendix S1 Tables S1 to S7. (DOCX)
