# Supplementary material for: Sustainable development of China’s aesthetic teaching in long-term policy changes
Source: PLoS One. 2025 Oct 14;20(10):e0334315. doi: 10.1371/journal.pone.0334315 (PMC12520396; doi:10.1371/journal.pone.0334315)
Supplement: Appendix S1 — (DOCX) [file pone.0334315.s001.docx]

**Supplementary Materials**

**Appendix S1: Tables S1 to S7**

**Table S1** Details of the sample policy documents (CCCPC and the State Council of PRC).

| **Number** | **Time** | **Name of policy document** | **Issuing institution** | **Reference source** |
| --- | --- | --- | --- | --- |
| 1 | 1999.06 | Decision on Deepening Education Reform and Comprehensively Promoting Quality Education | CCCPC and the State Council of PRC | https://ganxun.hue.edu.cn/2022/0331/c19774a137966/page.psp |
| 2 | 2012.09 | Opinions on Strengthening the Construction of the Teaching Team | The State Council of PRC | http://www.moe.gov.cn/jyb_xxgk/moe_1777/moe_1778/201209/t20120907_141772.html |
| 3 | 2013.11 | Decision on Several Major Issues Concerning the Comprehensive Deepening Reform | CCCPC | http://www.moe.gov.cn/jyb_xxgk/moe_1777/moe_1778/201311/t20131115_159502.html |
| 4 | 2015.01 | Opinions of the Central Committee of the Communist Party of China on the Prosperity and Development of Socialist Literature and Art | CCCPC | https://www.rmzxb.com.cn/c/2016-08-22/992366_3.shtml |
| 5 | 2015.9 | Opinions on Comprehensively Strengthening and Improving School Aesthetic Education Work | General Office of the State Council | http://www.moe.gov.cn/jyb_xxgk/moe_1777/moe_1778/201509/t20150928_211095.html |
| 6 | 2017.1 | Opinions on Deepening the Reform of the Professional Title System | CCCPC and the State Council of PRC | https://www.gov.cn/xinwen/2017-01/08/content_5157909.htm |
| 7 | 2017.4 | Medium- and Long-Term Youth Development Plan (2016-2025) | CCCPC and the State Council of PRC | https://www.gov.cn/zhengce/2017-04/13/content_5185555.htm#1 |
| 8 | 2018.01 | Opinions on Comprehensively Deepening the Reform of Teacher Team Construction in the New Era | CCCPC and The State Council of PRC | http://www.gov.cn/zhengce/2018-01/31/content_5262659.htm |
| 9 | 2018.02 | Guiding Opinions on Promoting the Reform of Talent Evaluation Mechanism by Classification | CCCPC and the State Council of PRC | https://www.gov.cn/gongbao/content/2018/content_5271732.htm |
| 10 | 2019.2 | Modernization of Education in China 2035 | CCCPC and the State Council of PRC | http://www.moe.gov.cn/jyb_xwfb/s6052/moe_838/201902/t20190223_370857.html |
| 11 | 2019.2 | Implementation Plan for Accelerating the Modernization of Education (2018-2022) | CCCPC and the State Council of PRC | http://www.moe.gov.cn/jyb_xwfb/s6052/moe_838/201902/t20190223_370859.html |
| 12 | 2019.6 | Guiding Opinions on Promoting the Reform of Education Methods in Ordinary High Schools in the New Era | The State Council of PRC | https://www.gov.cn/xinwen/2019-06/19/content_5401610.htm |
| 13 | 2019.7 | Opinions on Deepening Education and Teaching Reform and Comprehensively Improving the Quality of Compulsory Education | CCCPC and the State Council of PRC | http://www.moe.gov.cn/jyb_xwfb/gzdt_gzdt/s5987/201907/t20190708_389403.html |
| 14 | 2020.10 | Opinions on Comprehensively Strengthening and Improving the Aesthetic Education in Schools in the New Era | CCCPC and the State Council of PRC | http://www.moe.gov.cn/jyb_xxgk/moe_1777/moe_1778/202010/t20201015_494794.html |
| 15 | 2020.10 | Overall Plan for Deepening the Reform of Education Evaluation in the New Era | CCCPC and the State Council of PRC | http://www.moe.gov.cn/jyb_xxgk/moe_1777/moe_1778/202010/t20201013_494381.html |
| 16 | 2021.06 | Opinions on Deepening the Reform of State-owned Literature and Art Troupes | CCCPC and the State Council of PRC | https://www.gov.cn/gongbao/content/2021/content_5621193.htm |

**Table S2** Details of the sample policy documents (Ministries and commissions).

| **Number** | **Time** | **Name of policy document** | **Issuing institution** | **Reference source** |
| --- | --- | --- | --- | --- |
| 1 | 1989.11 | The Master Plan for Art Education in Schools Nationwide (1989-2000) (hereinafter referred to as the "Master Plan") | State Education Commission | https://kns.cnki.net/KXReader/Detail?invoice=c%2F46TQN5ossdHZpGRt%2F70isTE5z7i%2Bzci4PtCXbYWo%2BvB%2Fdbu4aCNei8H3AFvRaULbGBnLrmOzbBUWJbbdeiJ4C7YyTrmDrZWmVVIqnezYWIqhwpzBqHbQMLkUOzZTCTS8xBHFEeve4SmNFqe8yvdAZiljXx78nS76at3Mko5ls%3D&DBCODE=CJFD&FileName=ZYJA198903000&TABLEName=cjfdlast2018&nonce=50777DB1283F435FA13B80B6448189E5&uid=&TIMESTAMP=1681087820123 |
| 2 | 1993.02 | Outline of China's Education Reform and Development |  | http://jyt.jiangxi.gov.cn/art/2005/12/13/art_30381_1480737.html |
| 3 | 1996.07 | Opinions on Strengthening Art Education in National Colleges and Universities |  | https://www.cnki.com.cn/Article/CJFDTotal-ZYJA199705004.htm |
| 4 | 1997.05 | Opinions of the State Education Commission on Strengthening Art Education in Schools |  | https://kns.cnki.net/kcms2/article/abstract?v=lh5gWj_uM90tyaezIyeBaMZCHXcQa9PG0QGOons6wq0MWYpAkI0kF23QBX2n1EZPIDCxmQiwSY7vXVzLDW5IKKb-jYy68MS3UywEz9puWRjQk7LRtKXGryD6d0mBHX5x&uniplatform=NZKPT |
| 5 | 1998.12 | Action Plan for Revitalizing Education in the 21st Century | Ministry of Education of PRC | https://wenku.so.com/d/65ce118a0242f44b5224d58be71cbe67 |
| 6 | 2000.2 | Notice of the General Office of the Ministry of Education on Conducting National Rural School Art Education Experiments |  | http://www.moe.gov.cn/srcsite/A17/moe_794/moe_919/200002/t20000213_80447.html |
| 7 | 2002.5 | Work Regulations for School Art Education |  | http://www.moe.gov.cn/srcsite/A02/s5911/moe_621/200207/t20020725_81854.html |
| 8 | 2002.5 | Notice on the National Development Plan for School Art Education (2001-2010) |  | http://www.moe.gov.cn/srcsite/A17/moe_794/moe_795/200205/t20020513_80694.html |
| 9 | 2004.11 | Outline of China's Education Development and Reform (1990-2000) |  | https://wenku.baidu.com/view/d3942e5359cfa1c7aa00b52acfc789eb162d9e56.html?_wkts_=1681880033532 |
| 10 | 2005.02 | National Experimental Plan for the Reform and Development of Art Education in Rural Schools |  | http://www.moe.gov.cn/srcsite/A17/moe_794/moe_919/200002/t20000213_80447.html |
| 11 | 2008.9 | Opinions of the Ministry of Education on Further Strengthening Art Education in Primary and Secondary Schools |  | http://www.moe.gov.cn/srcsite/A17/moe_794/moe_795/200809/t20080908_80591.html |
| 12 | 2008.09 | Opinions on Further Strengthening the Work of Cultural and Art Archives | Ministry of Culture and Tourism of PRC | https://zwgk.mct.gov.cn/zfxxgkml/zcfg/gfxwj/202012/t20201204_906134.html |
| 13 | 2010.07 | Outline of the National Medium- and Long-Term Education Reform and Development Plan (2010-2020) | Ministry of Education of PRC | http://www.moe.gov.cn/srcsite/A01/s7048/201007/t20100729_171904.html |
| 14 | 2011.04 | Notice on the Implementation of the "Sports and Art 2+1 Project" in Primary and Secondary Schools during the Compulsory Education Stage |  | http://www.moe.gov.cn/srcsite/A17/s7059/201104/t20110426_171748.html |
| 15 | 2014.01 | Several Opinions on Promoting the Development of School Art Education |  | http://www.moe.gov.cn/srcsite/A17/moe_794/moe_795/201401/t20140114_163173.html |
| 16 | 2014.12 | Excellent Teacher Training Program |  | http://www.moe.gov.cn/srcsite/A10/s7011/201408/t20140819_174307.html |
| 17 | 2015.05 | Evaluation Measures for the Artistic Quality of Primary and Secondary School Students | Ministry of Education of PRC | http://www.moe.gov.cn/srcsite/A17/moe_794/moe_795/201506/t20150618_190674.html |
| 18 | 2015.05 | Self-evaluation Measures for Artistic Education Work in Primary and Secondary Schools |  |  |
| 19 | 2015.05 | Annual Report Measures for the Development of Artistic Education in Primary and Secondary Schools |  |  |
| 20 | 2015.12 | Notice on the Management Measures for National Art and Science Planning Projects | Ministry of Culture and Tourism of PRC | https://www.gov.cn/gongbao/content/2003/content_62098.htm |
| 21 | 2017.09 | Notice of the Ministry of Education on the Establishment of Chinese Excellent Culture and Art Inheritance Schools in Primary and Secondary Schools across the Country | Ministry of Education of PRC | http://www.moe.gov.cn/srcsite/A17/moe_794/moe_628/201710/t20171017_316628.html |

**Table S2** Details of the sample policy documents (Ministries and commissions). (Continued)

| **Number** | **Time** | **Name of policy document** | **Issuing institution** | **Reference source** |
| --- | --- | --- | --- | --- |
| 22 | 2017.10 | Guiding Outline of Comprehensive Practical Activities Curriculum for Primary and Secondary Schools | Ministry of Education of PRC | http://www.moe.gov.cn/srcsite/A26/s8001/201710/t20171017_316616.html |
| 23 | 2017.11 | Notice on the Management Measures for Part-time Teachers of School Physical and Aesthetic Education |  | http://www.moe.gov.cn/srcsite/A17/moe_794/moe_795/201711/t20171102_318281.html |
| 24 | 2018.03 | Notice on the Action Plan for Revitalizing Teacher Education (2018-2022) |  | http://www.moe.gov.cn/srcsite/A10/s7034/201803/t20180323_331063.html |
| 25 | 2018.04 | Notice on Carrying out the 2018 Elegant Art Campus Activity |  | http://www.moe.gov.cn/srcsite/A17/moe_794/moe_628/201804/t20180419_333675.html |
| 26 | 2018.10 | Opinions on Implementing the Excellent Teacher Training Plan 2.0 |  | http://www.moe.gov.cn/jyb_xwfb/s5147/201810/t20181011_351107.html |
| 27 | 2018.11 | Notice Regarding the Study and Implementation of the Important Reply Letter from the Jinping Xi General Secretary to the Senior Professor of the Central Academy of Fine Arts |  | http://www.moe.gov.cn/srcsite/A17/s7059/201808/t20180831_346877.html |
| 28 | 2018.11 | Notice on "Comprehensive Support for Art Education, Art Class Becoming a New Favorite" |  | http://www.51jiaoyujia.com/encyclopedia/consult_detail?id=5264 |
| 29 | 2019.04 | Opinions on Effectively Strengthening the Aesthetic Education Work in Higher Education Institutions in the New Era |  | http://www.moe.gov.cn/srcsite/A17/moe_794/moe_624/201904/t20190411_377523.html |
| 30 | 2019.06 | Notice on the Plan for Conducting Sports and Aesthetic Education Infiltration Behavior |  | http://www.moe.gov.cn/jyb_xwfb/gzdt_gzdt/s5987/201906/t20190625_387596.html |
| 31 | 2020.08 | Notice on the Establishment of the First National College Aesthetic Education Teaching Guidance Committee |  | http://www.moe.gov.cn/srcsite/A17/moe_794/moe_624/202008/t20200814_478031.html |
| 32 | 2020.09 | Opinions on Strengthening the Construction of Rural Teachers in the New Era | Six departments including the Ministry of Education | http://www.moe.gov.cn/srcsite/A10/s3735/202009/t20200903_484941.html |
| 33 | 2021.01 | Guiding Opinions on Strengthening the Construction Reform of the Teaching Staff in Universities in the New Era |  | http://www.moe.gov.cn/srcsite/A10/s7151/202101/t20210108_509152.html |
| 34 | 2021.06 | Art Creation Plan for the 14th Five Year Plan | Ministry of Culture and Tourism of PRC | https://www.mct.gov.cn/whzx/whyw/202107/t20210713_926402.htm |
| 35 | 2021.09 | Guiding Opinions on Further Strengthening and Improving the Enrollment Work of Art Majors in Ordinary Higher Education Institutions | Ministry of Education of PRC | http://www.moe.gov.cn/srcsite/A15/moe_776/s3109/202109/t20210923_566071.html |
| 36 | 2022.02 | Notice of the General Office of the Ministry of Culture and Tourism on Doing a Good Job in the Management of Off campus Training in Cultural and Art Courses | Ministry of Culture and Tourism of PRC | https://zwgk.mct.gov.cn/zfxxgkml/kjjy/202202/t20220215_931020.html |
| 37 | 2022.04 | Guiding Opinions on Promoting the High-Quality Development of Cultural and Art Vocational Education in the New Era |  | https://zwgk.mct.gov.cn/zfxxgkml/kjjy/202204/t20220424_932643.html |
| 38 | 2022.05 | New Era Basic Education Strong Teacher Program | Eight departments including the Ministry of Education | https://www.moe.gov.cn/srcsite/A10/s7034/202204/t20220413_616644.html |
| 39 | 2022.11 | Notice on the Guidelines for Public Art Courses in Higher Education Institutions | Ministry of Education of PRC | http://www.moe.gov.cn/jyb_xwfb/gzdt_gzdt/s5987/202212/t20221201_1010266.html |
| 40 | 2023.01 | Notice on the Implementation of the 2023 National Action Plan for Improving the Quality and Excellence of Cultural and Art Vocational Education and Tourism Vocational Education | Ministry of Culture and Tourism of PRC | https://zwgk.mct.gov.cn/zfxxgkml/kjjy/202301/t20230128_938791.html |

**Table S3** Dimension analysis table of the content elements of the policy on the construction of teaching staff in aesthetic education.

| **Policy content** | **Orientation of goal** | **Content elements** | **Text sample** | **Text source** |
| --- | --- | --- | --- | --- |
| Professional development | Providing activities and training services (including pre-post and introductory education) is helpful to the professional growth of teachers to enable the continuous evolution and enrichment of the internal structure of teachers. | Provide funds for training, exchange, study, cooperation, publicity, etc. | Improve the professional level of teachers. Improve the training system, make training plans, optimize the structure of teachers, and improve the professional level and teaching ability of teachers. Cultivate education and teaching backbone, double-professionally-titled teachers, academic leaders and principals through research and training, academic exchanges, project funding and other means, to train a group of famous teachers and discipline leaders. | Outline of China's Medium- and Long-Term Plan for Education Reform and Development |
| Post management | Provisions on post establishment, teacher resource allocation, replenishment mechanism, post flow, etc. | Establishment standards, flow mechanisms, etc. | The education administrative department shall set up a special organization to administer school art education, appoint special personnel to take charge of the administration of school art education, and appoint full-time or part-time cadres to take charge of the administration. | Development Plan for Art Education in Schools Nationwide |
| Benefits | Regulations on teachers' salaries, social security, and sanctions. | Subsidies, social security, preferential policies, incentives, etc. | Improve the status and benefits of teachers. Constantly improving the working, learning, and living conditions of teachers, ensure that the mean salary of teachers is not lower than or higher than the mean salary of civil servants according to law, and gradually raise it. | Outline of China's Medium- and Long-Term Plan for Education Reform and Development |
| Quality assessment | Evaluation of teachers' professional titles, professional standards, and teaching effects. | Supervision, diagnosis, feedback, inspection, evaluation, etc. | Improve the evaluation system of aesthetic education in higher education institutions, bring the effect of aesthetic education into the evaluation index system of talent training in higher education institutions, pay attention to the process and effect evaluation, and play to the role of expert organizations and social organizations in the evaluation of aesthetic education. | Opinions on Effectively Strengthening the Aesthetic Education in Higher Education Institutions in the New Era |

**Note:** (1). The ten elements of teacher policy constructed by the World Bank in 2012 (Pesambili et al. 2022; Jiang and Zhang 2017) were as follows: “Entry and Retention Policy”, “Pre-post Training Policy”, “Recruitment and Employment Policy”, “Post Management Policy”, “Professional Development Policy”, “Supervision and Evaluation Policy”, “Remuneration and Benefit Policy”, “Retirement and Welfare Policy”, “Teachers' Discourse Right Policy”, “School Leadership Policy”. (2). According to the interpretation of the ten elements of teacher policy by the World Bank and combined with the actual content of the policy on construction of China's teaching staff in aesthetic education, this study finally listed four dimensions: professional development, post management, benefits, and quality assessment. The "Entry and Retention Policy", "Pre-post Training Policy", "Recruitment and Employment Policy" and “Professional Development Policy” were combined into "Professional Development". The "Remuneration and Benefit Policy" and "Welfare Policy" were combined into "Benefits". The "Supervision and Evaluation Policy", "Teacher Voice Policy", and "School Leadership Policy" were merged into "Quality Assessment" with a focus on monitoring, diagnosis, feedback, and inspection processes and effectiveness evaluation. Since the "retirement rule" was not clearly reflected in our aesthetic education policy, it was deleted in this study.

Pesambili, J.C., Sayed, Y., Stambach, A. 2022. The World Bank’s construction of teachers and their work: A critical analysis. *International Journal of Educational Development* 92:102609. https://doi.org/10.1016/j.ijedudev.2022.102609.

Jiang, X., Zhang, S 2017. The framework and feature of World Bank’s teacher policy and its enlightenments. *Primary & Secondary Schooling Abroad* 36(4):47-53. https://doi.org/10.3969/j.issn.1007-8495.2017.04.008.

**Table S4** Text content analysis coding table of the policies on the construction of teaching staff in aesthetic education (CCCPC, the State Council of PRC).

| **Policy instruments** | **Policy content elements** | **Code** | **Subtotal** | **Percentage (%)** |
| --- | --- | --- | --- | --- |
| Command instruments | Professional development | 1-3-3-2; 2-2-4; 2-3-8; 5-3-1; 5-4-13; 8-3-2; 8-4-1; 9-4-11-1; 10-7-2; 11-1; 11-6; 14-4-13*; 15-2-4-17 | 13 | 11.61 |
|  | Benefits | 11-5-2; 14-5-17* | 2 | 1.79 |
|  | Post management | 1-3-3-1; 1-4-2; 6-2-4; 13-4-13 | 4 | 3.57 |
|  | Quality assessment | 8-4-4*; 9-4-11-2; 9-4-12-1; 10-7-1; 13-6-25; 14-5-18*; 15-2-2-8; 15-2-3-9; 15-2-3-10*; 15-2-3-12 | 10 | 8.93 |
| Incentive instruments | Professional development | 2-4-15*; 4-5-20*; 4-6-24; 5-4-2; 12-2-6* | 5 | 4.46 |
|  | Benefits | 2-5-19; 7-2-6-5; 10-7-3; 12-3-6; 13-4-14; 14-4-13*; 15-2-3-10* | 7 | 6.25 |
|  | Post management | 15-2-5-22 | 1 | 0.89 |
|  | Quality assessment | —— | 0 | 0 |
| Capability building instruments | Professional development | 1-3-4-2; 1-3-5-2; 2-3-8; 2-3-10*; 2-4-14*; 3-5-20*; 3-6-24; 4-5-20; 5-3-3*; 5-3-4; 5-3-5*; 5-3-6; 5-3-7; 5-3-8; 7-2-6-2; 7-2-6-3; 8-3-1*; 8-3-4; 8-4-3*; 11-9; 13-4-12; 14-4-15*; 16-2-6; 16-3-3 | 24 | 21.43 |
|  | Benefits | 1-3-3-4 | 1 | 0.89 |
|  | Post management | 5-3-3*; 5-3-5* | 2 | 1.79 |
|  | Quality assessment | —— | 0 | 0 |
| System changing instruments | Professional development | 1-3-1-4-; 1-3-3-3; 1-3-4-4; 1-3-5-1; 2-3-10*; 2-3-12; 2-4-14*; 2-4-15*; 3-5-20*; 4-5-20*; 5-3-5*; 8-2-3; 11-3; 11-5-3; 14-3-9; 14-4-15*; 14-5-17*; 14-5-18* | 18 | 16.07 |
|  | Benefits | —— | 0 | 0 |
|  | Post management | 2-4-13; 10-2-3-11*; 11-5-1; 13-2-6* | 4 | 3.57 |
|  | Quality assessment | 2-5-20; 5-3-10; 5-4-3; 8-3-1*; 8-4-4*; 13-5-19; 14-5-18* | 7 | 6.25 |
| Exhortation instruments | Professional development | 2-4-14*; 5-3-2; 5-3-9; 7-2-6-1; 7-2-10-3-5; 7-2-10-3-8; 8-3-1*; 8-4-3*; 14-3-12; 14-4-14; 14-4-15; 14-5-17*; 16-2-7 | 13 | 11.61 |
|  | Benefits | —— | 0 | 0 |
|  | Post management | 14-4-13* | 1 | 0.89 |
|  | Quality assessment | —— | 0 | 0 |

Note: (1) The "*" indicated that the same code corresponds to more than one policy instrument, so repeated code was marked with "*". (2) This was attributed to limited space, so only part of the coding information was provided in this paper.

**Table S5** Text content analysis coding table of the policies on the construction of teaching staff in aesthetic education (Ministries and commissions including the Ministry of Education of PRC and Ministry of Culture and Tourism of PRC).

| **Policy instruments** | **Policy content elements** | **Code** | **Subtotal** | **Percentage (%)** |
| --- | --- | --- | --- | --- |
| Command instruments | Professional development | 1-2-3-1; 1-4-2-1*; 1-4-2-2; 1-4-4-2; 2-4-35; 2-5-43; 3-5-1; 8-3-4-4; 11-5-13; 13-53-3; 13-53-4; 13-55-1; 13-60-1; 14-4-2; 14-4-4; 16-5-4; 16-6-1; 19-2; 21-5-3; 22-4-2-4; 23-3-12*; 24-3-1; 24-3-2; 24-3-4; 24-4-2; 26-3-1; 29-3-2; 32-4-8*; 32-6-16*; 32-7-17; 33-2-4; 33-2-5*; 33-7-18*; 34-1; 34-2; 35-2-1-2; 37-3-8; 37-3-10; 37-4-15; 37-5-16; 38-2-2; 40-1 | 42 | 14.89 |
|  | Benefits | 1-4-4-3; 2-5-39; 2-5-42; 2-5-44; 7-4-14-2; 8-3-3-4*; 8-3-5-2; 9-5-43; 9-5-44; 11-4-11*; 13-54-1; 15-2-6-2; 15-4-13-2; 30-6-2; 37-4-14*; 37-5-17; 38-2-14; 38-3-3* | 18 | 6.38 |
|  | Post management | 7-4-13-1; 8-3-3-1; 8-4-1*; 8-4-2; 11-4-9; 16-6-2*; 23-2-5; 23-2-7-1; 23-3-11; 23-4-13*; 23-4-14*; 23-4-15; 29-4-1; 32-3-5*; 32-3-6*; 32-3-7*; 33-4-8*; 33-4-11; 33-6-17; 38-2-10; 38-2-11; 38-2-12; 39-9-2 | 23 | 8.16 |
|  | Quality assessment | 1-4-2-1*; 1-4-3-9; 4-4-3*; 15-4-13-1; 18-3; 18-5; 20-2-6-4*; 22-5-3-2; 23-4-13*; 24-4-3; 26-3-3; 26-4-4; 29-4-4*; 32-6-14*; 33-2-5*; 33-3-6*; 33-4-10; 36-3-1; 37-5-18; 38-2-13; 38-3-1* | 21 | 7.45 |
| Incentive instruments | Professional development | 1-4-4-4; 4-4-3*; 7-5-18; 13-55-3; 16-6-2*; 16-7-2*; 23-5-18; 32-4-8*; 32-4-10*; 32-7-19; 32-8-20; 33-3-7*; 33-7-18*; 37-5-19 | 14 | 4.96 |
|  | Benefits | 1-4-4-1; 3-5-2*; 4-6-3; 7-5-17; 8-3-3-4*; 9-5-45; 20-2-5-7; 22-5-3-1; 32-7-18; 32-9-23; 33-5-15; 33-7-19; 39-9-4 | 13 | 4.61 |
|  | Post management | 11-4-10*; 23-4-16; 32-3-5*; 32-4-8*; 32-6-14*; 33-5-14 | 6 | 2.13 |
|  | Quality assessment | —— | 0 | 0 |
| Capability building instruments | Professional development | 1-2-3-2; 1-4-3-3*; 1-4-3-4; 1-4-3-5; 1-4-3-6; 1-4-3-7; 2-5-41-1; 4-4-2; 4-6-1*; 4-6-2; 5-2-8; 7-4-14-1; 8-3-3-3; 8-3-5-1; 9-5-40; 11-4-11*; 13-53-1; 13-53-2*; 14-4-1*; 15-4-11*; 16-3-2; 16-6-2*; 16-7-2*; 22-3-1-4; 22-5-1-1; 23-3-12*; 23-5-19; 24-3-3; 24-3-5*; 24-3-6; 24-3-8; 26-3-2; 26-3-7; 26-4-2; 26-4-3; 29-3-1*; 29-3-4; 30-3-4; 32-5-11*; 32-5-12; 32-5-13; 32-6-16*; 33-3-6*; 33-4-8*; 33-7-18*; 37-3-11; 37-3-12*; 37-4-14*; 38-2-6; 38-2-7; 38-3-3*; 39-9-1* | 52 | 18.44 |
|  | Benefits | 3-5-2 | 1 | 0.36 |
|  | Post management | 15-2-6-1*; 23-2-7-2; 24-3-7; 32-3-6*; 39-9-1* | 5 | 1.77 |
|  | Quality assessment | —— | 0 | 0 |
| System changing instruments | Professional development | 1-4-3-3*; 1-4-3-7; 1-4-3-8; 2-5-41-2; 8-3-5-3; 11-2-5; 15-2-4-2; 16-3-1; 20-2-5-6; 21-3-1; 21-5-1; 22-4-2-2; 23-4-14*; 24-3-5*; 26-3-4; 26-3-6; 29-2-3; 29-4-3*; 32-5-11*; 37-3-9; 38-2-4; 38-2-5; 38-2-8; 38-2-9; 38-3-3*; 39-5; 39-9-3 | 27 | 9.57 |
|  | Benefits | —— | 0 | 0 |
|  | Post management | 1-4-2-3; 4-4-1; 4-6-1*; 5-2-10; 8-3-4-1; 8-4-1*; 9-5-42; 13-55-2; 14-4-3; 15-2-6-1*; 15-4-11*; 23-5-17; 32-3-5*; 32-4-9*; 33-4-9; 37-3-12*; 39-8 | 17 | 6.03 |
|  | Quality assessment | 15-3-9; 15-3-10; 16-5-5; 18-6; 19-3; 24-3-10; 26-3-8; 29-3-1*; 29-4-4*; 33-4-12; 35-2-1-13; 38-3-2; 39-10 | 13 | 4.61 |
| Exhortation instruments | Professional development | 8-3-4-2; 9-5-41; 13-53-2*; 14-4-1*; 16-4-2; 16-6-2*; 20-2-6-4*; 23-2-6*; 24-3-9; 25-4-1; 26-3-5; 29-3-1*; 29-4-3*; 32-3-7*; 32-4-9*; 32-5-11*; 33-3-6*; 33-3-7*; 37-3-12*; 37-4-13; 37-4-14*; 38-2-3; 38-3-1* | 23 | 8.16 |
|  | Benefits | 2-5-46; 21-5-2 | 2 | 0.71 |
|  | Post management | 15-2-6-1*; 23-2-6*; 32-3-6*; 32-4-10*; 33-4-13 | 5 | 1.77 |
|  | Quality assessment | —— | 0 | 0 |

**Table S6** X-dimensional time series proportion of policy instruments for the construction of aesthetic education teacher team.

| CCCPC, the State Council of PRC (%) | | | | | |
| --- | --- | --- | --- | --- | --- |
| Stage division | Command instruments | Incentive instruments | Capability building instruments | System changing instruments | Exhortation instruments |
| First stage (1985-1990) | 0 | 0 | 0 | 0 | 0 |
| Second stage (1991-1995) | 0 | 0 | 0 | 0 | 0 |
| Third stage (1996-2000) | 30.00 | 0 | 30.00 | 40.00 | 0 |
| Fourth stage (2001-2005) | 0 | 0 | 0 | 0 | 0 |
| Fifth stage (2006-2010) | 0 | 0 | 0 | 0 | 0 |
| Sixth stage (2011-2015) | 10.81 | 13.51 | 37.84 | 29.73 | 8.11 |
| Seventh stage (2016-2020) | 35.48 | 12.90 | 12.90 | 22.58 | 16.13 |
| Eighth stage (2021-2025) | 0 | 0 | 66.67 | 0 | 33.33 |
| Ministries and commissions including the Ministry of Education of PRC and Ministry of Culture and Tourism of PRC (%) | | | | | |
| Stage division | Command instruments | Incentive instruments | Capability building instruments | System changing instruments | Exhortation instruments |
| First stage (1985-1990) | 36.84 | 10.53 | 31.58 | 21.05 | 0 |
| Second stage (1991-1995) | 62.50 | 0 | 12.50 | 12.50 | 12.50 |
| Third stage (1996-2000) | 15.38 | 23.08 | 38.46 | 23.08 | 0 |
| Fourth stage (2001-2005) | 41.67 | 16.67 | 16.67 | 16.67 | 8.33 |
| Fifth stage (2006-2010) | 50.00 | 12.50 | 18.75 | 12.50 | 6.25 |
| Sixth stage (2011-2015) | 32.43 | 8.11 | 16.22 | 29.73 | 13.51 |
| Seventh stage (2016-2020) | 31.91 | 12.77 | 23.40 | 18.09 | 13.83 |
| Eighth stage (2021-2025) | 42.25 | 9.86 | 15.49 | 21.13 | 11.27 |

**Table S7** The proportion of Y-dimension time series in the policy content of the construction of the aesthetic education teacher team.

| CCCPC, the State Council of PRC (%) | | | | |  |
| --- | --- | --- | --- | --- | --- |
| Stage division | Professional development | Benefits | Post management | Quality assessment |  |
| First stage (1985-1990) | 0 | 0 | 0 | 0 |  |
| Second stage (1991-1995) | 0 | 0 | 0 | 0 |  |
| Third stage (1996-2000) | 70.00 | 10.00 | 20.00 | 0 |  |
| Fourth stage (2001-2005) | 0 | 0 | 0 | 0 |  |
| Fifth stage (2006-2010) | 0 | 0 | 0 | 0 |  |
| Sixth stage (2011-2015) | 81.08 | 2.70 | 8.11 | 8.11 |  |
| Seventh stage (2016-2020) | 53.23 | 12.90 | 11.29 | 22.58 |  |
| Eighth stage (2021-2025) | 100.00 | 0.00 | 0.00 | 0 |  |
| Ministries and commissions including the Ministry of Education of PRC and Ministry of Culture and Tourism of PRC (%) | | | | |  |
| Stage division | Professional development | Benefits | Post management | Quality assessment |  |
| First stage (1985-1990) | 73.68 | 10.53 | 5.26 | 10.53 |  |
| Second stage (1991-1995) | 50.00 | 50.00 | 0 | 0.00 |  |
| Third stage (1996-2000) | 46.15 | 23.08 | 23.08 | 7.69 |  |
| Fourth stage (2001-2005) | 37.50 | 33.33 | 29.17 | 0 |  |
| Fifth stage (2006-2010) | 68.75 | 12.50 | 18.75 | 0 |  |
| Sixth stage (2011-2015) | 51.35 | 8.11 | 16.22 | 24.32 |  |
| Seventh stage (2016-2020) | 58.51 | 5.32 | 24.47 | 11.70 |  |
| Eighth stage (2021-2025) | 56.34 | 9.86 | 18.31 | 15.49 |  |
